# Nomogram for predicting the occurrence of progressive ischemic stroke: a single-center retrospective study

**DOI:** 10.1186/s40001-025-03171-5

**Published:** 2025-09-26

**Authors:** Yu Liu, Xiaoyu Xu, Yanlong Zhou, Bo Du, Yanbo Cheng, Yu Feng

**Affiliations:** 1 Department of Neurology, Affiliated Hospital of Xuzhou Medical University, Xuzhou, Jiangsu China; 2 Xuzhou Cancer Hospital, Xuzhou, Jiangsu China; 3 Fengxian People’s Hospital, Xuzhou, Jiangsu China

**Keywords:** Progressive stroke, Lipoprotein-associated phospholipase A2, Acute ischemic stroke, Nomogram, Prediction model, Risk factors

## Abstract

**Objectives:**

Progressive ischemic stroke (PIS) is a severe adverse cerebrovascular event that can occur shortly after an acute ischemic stroke (AIS).The clinical factors that predict PIS remain poorly understood. This study aims to develop a nomogram for predicting PIS following AIS.

**Methods:**

This study retrospectively analyzed clinical data from patients diagnosed with AIS at the Affiliated Hospital of Xuzhou Medical University between 2018 and 2021 who subsequently developed PIS. Risk factors associated with PIS were identified using univariate *logistic* regression, followed by stepwise multivariate *logistic* regression to construct a predictive model. The resulting model was then transformed into a nomogram, providing neurologists with a clinically practical tool for rapidly assessing the risk of PIS following AIS.

**Results:**

Among 580 patients with AIS, 14.31% developed progressive stroke within 14 days. The data set was split into a training set (70%) and a test set (30%). Univariate analysis identified ten indicators associated with progressive stroke, and multivariate logistic regression in the training set revealed four independent risk factors. A nomogram was developed using R software (version 4.3.2) to predict progressive stroke risk. The Model demonstrated strong performance, with ROC curve AUCs of 0.849 (training set) and 0.829 (test set). The *DeLong* test showed no significant difference between the data sets (*P* > 0.05), confirming robustness. The overall AUC was 0.974, and the *Hosmer–Lemeshow* test indicated good calibration (*P* = 0.887). The calibration plot’s mean absolute error was 0.012, and decision curve analysis confirmed the nomogram’s clinical utility. Internal validation showed close agreement between the training and test sets.

**Conclusions:**

The nomogram model appears to enhance the prediction of progressive stroke risk in patients with AIS, potentially supporting neurologists in making more informed and timely clinical decisions.

## Introduction

Ischemic stroke is one of the leading causes of mortality and disability worldwide[1]. The neurological impairments caused by stroke, whether transient or permanent, remain a significant challenge in stroke management and rehabilitation. Progressive ischemic stroke (PIS), a severe manifestation within the spectrum of ischemic strokes, occurs when arterial plaques rupture, leading to thrombosis that obstructs cerebral blood flow, often compounded by cardiogenic embolism and small artery occlusion. This results in localized ischemia and hypoxia, leading to tissue necrosis or softening. While some patients stabilize after an acute ischemic stroke (AIS), with gradual recovery of neurological functions thanks to medical treatment and natural healing processes, others deteriorate due to various risk factors, such as a history of type 2 diabetes mellitus (T2DM), advanced age, lipid metabolism disorders, and systemic inflammatory responses, leading to PIS [2–5].

Currently, there is no definitive criterion for PIS, but this study adopts a definition from prior research, identifying PIS as an increase of ≥ 2 points in the National Institutes of Health Stroke Scale score (NIHSS score) within 7 days of AIS onset [12]. Beyond the initial neurological deficits following an ischemic stroke, it is estimated that 25–33% of patients experience PIS [6], marked by worsening neurological symptoms within days after the initial event. Compared to patients without PIS, those with PIS face higher mortality rates, poorer functional outcomes, and more severe neurological deficits [7]. Previous studies have identified large artery atherosclerosis, diabetes mellitus, high low-density lipoprotein, hypertriglyceridemia, and smoking as independent risk factors for AIS [8]. Researchers like Markus Arnold have demonstrated that Lipoprotein(a) is an independent risk factor for recurrent atherosclerotic stroke in middle-aged and elderly patients [9], while Elkind et al. have shown that lipoprotein-associated phospholipase A2 (Lp-PLA2) is an independent predictor of recurrent stroke in AIS patients [10]. Recent findings from our research team confirm that factors such as age, T2DM, Lp-PLA2, and Lipoprotein(a) are independent predictors of long-term stroke recurrence in AIS patients, with the combined presence of Lp-PLA2 and Lipoprotein(a) showing high predictive power for stroke recurrence [11].

However, there is a scarcity of studies exploring the relationship between stroke-related target vessel disease, a history of hypertension, T2DM, advanced age, and lipid metabolism disorders with the occurrence of PIS following AIS. This study aims to identify independent risk factors for PIS in AIS patients and to develop a robust nomogram-based predictive model. This tool may aid clinicians in conducting preliminary assessments and initiating early interventions, which could potentially reduce the incidence of PIS and enhance long-term outcomes for these patients.

## Materials and methods

### Population characteristics

**Fig. 1:**
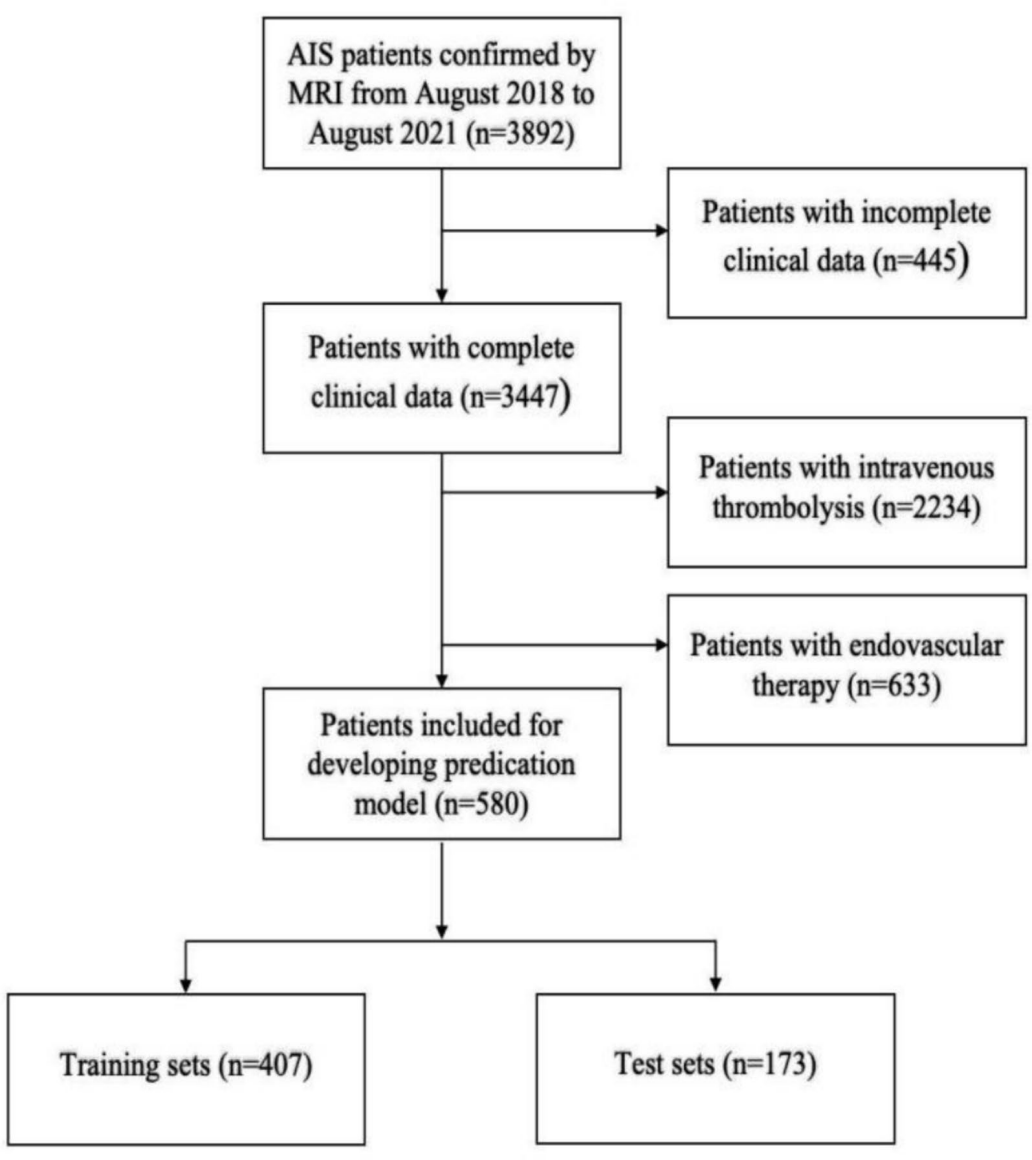
Flowchart summarizing the characteristics of the patients included in the study. Magnetic resonance imaging (MRI)

This retrospective study analysed the data of patients admitted with acute ischemic stroke (AIS) at the Affiliated Hospital of Xuzhou Medical University from August 2018 to August 2021. A total of 580 patients who met the study criteria were selected (Fig. 1). The study population consisted of individuals aged 18 years or older, who presented within 6 h of symptom onset and were diagnosed with AIS. All patients received treatment with clopidogrel and aspirin in the emergency department of our hospital, without the use of intravenous thrombolysis or endovascular therapy. The inclusion criterion of symptom onset to hospital admission within 6 h was primarily to align with the time definition of PIS, characterized by the progressive worsening of neurological deficits within 1 week and an increase in the NIHSS score by 2 points or More, despite receiving basic treatment after 6 h from stroke onset [12]. AIS was identified by the persistent or sometimes rapid development of neurological symptoms, accompanied by corresponding ischemic changes detected by magnetic resonance imaging (MRI) or computed tomography (CT) [13].

The study was conducted in compliance with the Declaration of Helsinki and was approved by the Ethics Committee of the Affiliated Hospital of Xuzhou Medical University. Informed consent was waived due to the retrospective nature of the study.

### Data collection

To minimize subjective judgment errors, two experienced neurologists independently assessed patients and documented relevant indicators. Patient data—including sex, age, contact information, and cerebrovascular risk factors (e.g., hypertension, diabetes, and heart disease)—as well as the duration from symptom onset to antiplatelet therapy initiation and prior medication use, were compiled. Hypertension was diagnosed according to the 2010 Chinese guidelines for hypertension management [14–17], and diabetes was diagnosed based on the 2013 Chinese guidelines for type 2 diabetes prevention and treatment [18]. Heart disease was defined as a known history or clinical evidence of any cardiac condition, including myocardial infarction, angina pectoris, congestive heart failure, or arrhythmia [19].

Upon emergency admission, venous blood samples were drawn from the elbow of all patients for routine blood tests, biochemical analysis, coagulation function assessment, and Lp-PLA2 quantification using immunoturbidimetry [20]. Neurological impairment severity was evaluated using the NIHSS score [21]. To confirm stroke subtypes, patients underwent magnetic resonance angiography (MRA) or computed tomography angiography (CTA), electrocardiography, and echocardiography. Acute ischemic stroke (AIS) cases were classified into subtypes according to the TOAST criteria [22], including large artery atherosclerosis (LAA), cardioembolism (CE), small artery occlusion (SAO), stroke of other determined etiology (SOD), and stroke of undetermined etiology (SUD).

Follow-up:

All instances of progressive ischemic stroke (PIS) were confirmed through clinical symptoms or neuroimaging data, including computed tomography (CT) or magnetic resonance imaging (MRI), as assessed by neurologists. During their hospital stay, patients received medications tailored to prevent stroke recurrence based on their specific stroke subtype. After a 7-day follow-up, patients were categorized into either the PIS group or the non-PIS group.

Statistical procedures:

Data were analyzed using SAS version 4. Continuous variables were described as mean ± standard deviation if normally distributed, with differences between groups evaluated using the *t* test. Non-normally distributed data were described as median (P25, P75), and differences were assessed using the Wilcoxon rank-sum test. Categorical variables were described as number of cases (%), with differences evaluated using the *χ*^*2*^ test. Ordinal variables were analyzed using the Kruskal–Wallis test or Spearman’s rank correlation, as appropriate. R version 4.3.2 was used to construct a multivariate logistic regression model. Univariate logistic regression analysis was employed to identify risk factors for PIS following AIS. Variables that achieved a significance level of *P* < 0.05 were subsequently incorporated into a multivariate logistic regression model using the forward likelihood ratio selection method, based on which a nomogram prediction model was generated. Model performance was evaluated using the ROC curve, with the area under the curve (AUC) serving as the metric. Model fit was assessed using calibration curves and the Hosmer–Lemeshow test. Decision curve analysis (DCA) was conducted to evaluate the model’s clinical utility. A *P* value < 0.05 was considered statistically significant.

## Results

### Lp-PLA2 value significant increased in the progressive ischemic stroke

A total of 580 eligible patients were included in this study, and 83 patients experienced progressive stroke (14.31%). Among them, 398 were male and 182 were female, aged between 23 and 82 years, with a mean age of (62.51 ± 10.53) years. To establish and evaluate the prediction Model, the initial data were randomly divided into training and test data sets in a 7:3 ratio. The training data set comprised 407 patients, of whom 59 experienced progressive stroke (incidence of 14.50%); the test set included 173 patients, with 24 experiencing progressive stroke (incidence of 13.87%). The baseline data of the training cohort revealed significant differences in LAA, hypertension (EH), T2DM, NIHSS score, age, Lipoprotein(a), Apolipoprotein B, neutrophils, lymphocyte, and Lp-PLA2 between the recurrence and non-recurrence groups (*P* < 0.05, Table 1), indicating that these variables were sensitive indicators for PIS. There were no significant differences in gender, white blood cell, total cholesterol, LDL-cholesterol (LDL-C), and other variables between the PIS group and the Non-PIS group *(P* > 0.05, Table 1).

**Table 1:** Basic clinical characteristics between non-PIS groups and PIS groups in patients with acute ischemic stroke (AIS)

Apolipoprotein B	0.84(0.70,1.04)	0.97(0.79,1.18)	2.820	**0.005**
Total cholesterol	4.39(3.81,5.16)	4.70(3.90,5.55)	1.793	**0.073**
White blood cell	6.15(5.20,7.25)	6.60(5.50,8.00)	1.768	**0.077**
Neutrophils	3.97(3.23,4.87)	4.52(3.49,5.98)	2.901	**0.004**
Lymphocyte	1.60(1.20,1.90)	1.30(1.10,1.60)	-3.256	**0.001**
Percentage of monocytes	0.35(0.28,0.45)	0.38(0.32,0.44)	1.708	**0.088**
Percentage of eosinophils	0.09(0.05,0.17)	0.10(0.06,0.13)	0.031	**0.975**
Percentage of basophils	0.01(0.00,0.02)	0.01(0.00,0.02)	0.780	**0.436**
Red blood cell	4.63(4.25,4.89)	4.58(4.16,4.90)	− 0.266	**0.790**
Hemoglobin	142.00(131.00,151.00)	140.00(131.00,151.00)	− 0.809	**0.419**
Platelet	202.50(175.50,236.00)	206.00(180.00,238.00)	0.284	**0.776**
Lp-PLA2	168.50(107.00,293.00)	406.00(226.00,490.00)	7.068	** < 0.0001**
Procalcitonin	0.22(0.19,0.25)	0.21(0.18,0.27)	− 0.332	**0.740**
Mean platelet volume	10.60(9.80,11.40)	10.20(9.60,11.30)	− 1.539	**0.124**
Large platelet ratio	29.60(23.70,36.70)	27.90(23.90,35.00)	− 1.169	**0.242**

LAA, large artery atherosclerosis; EH, Hypertension; T2DM, Type 2 diabetes mellitus; NIHSS score, National Institutes of Health Stroke Scale score

LAA, EH, T2DM, NIHSS score, age, Lipoprotein(a), Apolipoprotein B, Neutrophils, Lymphocyte, and Lp-PLA2 were statistically different between the two groups (*P* < 0.05). No other variables were statistically significant (*P* > 0.05)

### T2DM, LAA, age, and Lp-PLA2 were independent risk factors for progressive stroke in patients

**Table:** 

Basic clinical characteristics	Non-PIS (N = 348)	PIS (N = 59)	χ^2^/Z	*P* value
Gender			1.351	**0.245**
Male	239(68.68%)	36(61.02%)		
Female	109(31.32%)	23(38.98%)		
LAA			14.296	** < 0.0001**
No	187(53.74%)	16(27.12%)		
Yes	161(46.26%)	43(72.88%)		
EH			5.2 87	**0.022**
No	137(39.37%)	14(23.73%)		
Yes	211(60.63%)	45(76.27%)		
T2DM			16.722	** < 0.0001**
No	275(79.02%)	32(54.24%)		
Yes	73(20.98%)	27(45.76%)		
NIHSS score			6.304	**0.012**
1	296(85.06%)	42(71.19%)		
2	46(13.22%)	15(25.42%)		
3	6(1.72%)	2(3.39%)		
Age	61.00(54.00,71.00)	67.00(63.00,73.00)	4.180	** < 0.0001**
Homocysteine	13.72(11.00,17.51)	13.88(10.78,17.02)	0.184	**0.854**
LDL-C	2.58(2.10,3.30)	3.01(2.23,3.58)	1.946	**0.052**
HDL-C	1.14(0.94,1.34)	1.10(0.93,1.37)	-0.511	**0.609**
Triglyceride	1.29(0.95,1.86)	1.47(1.13,1.87)	1.675	**0.094**
Lipoprotein(a)	204.00(129.00,304.50)	224.00(172.00,443.00)	2.056	**0.040**

To construct a comprehensive prediction model, univariate logistic regression analysis was conducted on variables that differed between the two groups of progressive stroke. The analysis revealed that LAA, EH, T2DM, NIHSS score, Age, Apolipoprotein B, Lymphocyte, and Lp-PLA2 exhibited significant statistical significance (*P* < 0.05, Table 1). Consequently, a multivariate logistic regression prediction model was developed using stepwise regression for the meaningful variables, which indicated that T2DM, LAA, Age, and Lp-PLA2 were independent risk factors for progressive stroke (*OR* > 1, and *P* < 0.05, Table 2).

**Table 2:** Statistical independent risk factors among the risk factors associated with progressive stroke

Parameters	*β*	StdError	OR(95%CI)	Wald *x*^2^	*P* value
NIHSS score	0.660	0.270	1.94(1.12,3.25)	2.447	0.014
Age	0.312	0.077	1.37(1.18,1.60)	4.043	< 0.0001
Lipoprotein(a)	0.001	0.001	1.00(1.00,1.00)	1.613	0.107
Apolipoprotein B	1.570	0.565	4.81(1.60,14.70)	2.780	0.005
Neutrophils	0.003	0.013	1.00(0.95,1.03)	0.212	0.832
Lymphocyte	-0.854	0.295	0.43(0.23,0.74)	-2.890	0.004
Lp-PLA2	0.692	0.100	2.00(1.65,2.45)	6.924	< 0.0001
LAA					
No	Reference	–	–	–	–
Yes	1.138	0.312	3.12(1.73,5.91)	3.649	< 0.0001
EH					
No	Reference	–	–	–	–
Yes	0.736	0.325	2.09(1.13,4.08)	2.263	0.024
T2DM					
No	Reference	–	–	–	–
Yes	1.156	0.293	3.18(1.78,5.64)	3.952	< 0.0001

LAA, large artery atherosclerosis; EH, Hypertension; T2DM, Type 2 diabetes mellitus; NIHSS score, National Institutes of Health Stroke Scale score

T2DM, LAA, Age, and Lp-PLA2 were independent risk factors for progressive stroke in patients (OR > 1, P < 0.05)

### Effciency of the nomogram model was evaluated in training and test cohorts

**Fig. 2:**
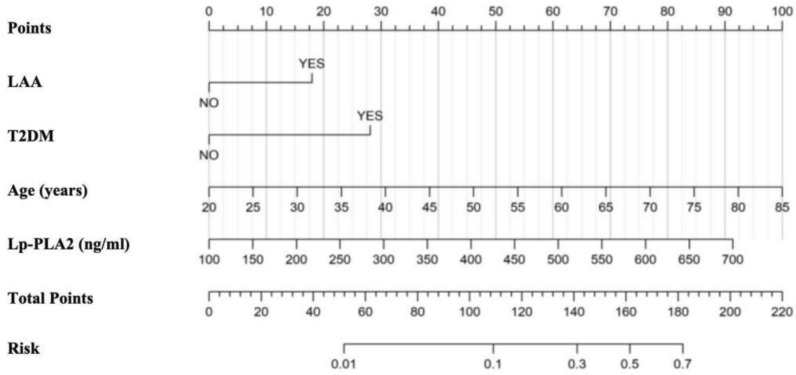
Progressive stroke risk prediction nomogram

**Fig. 3:**
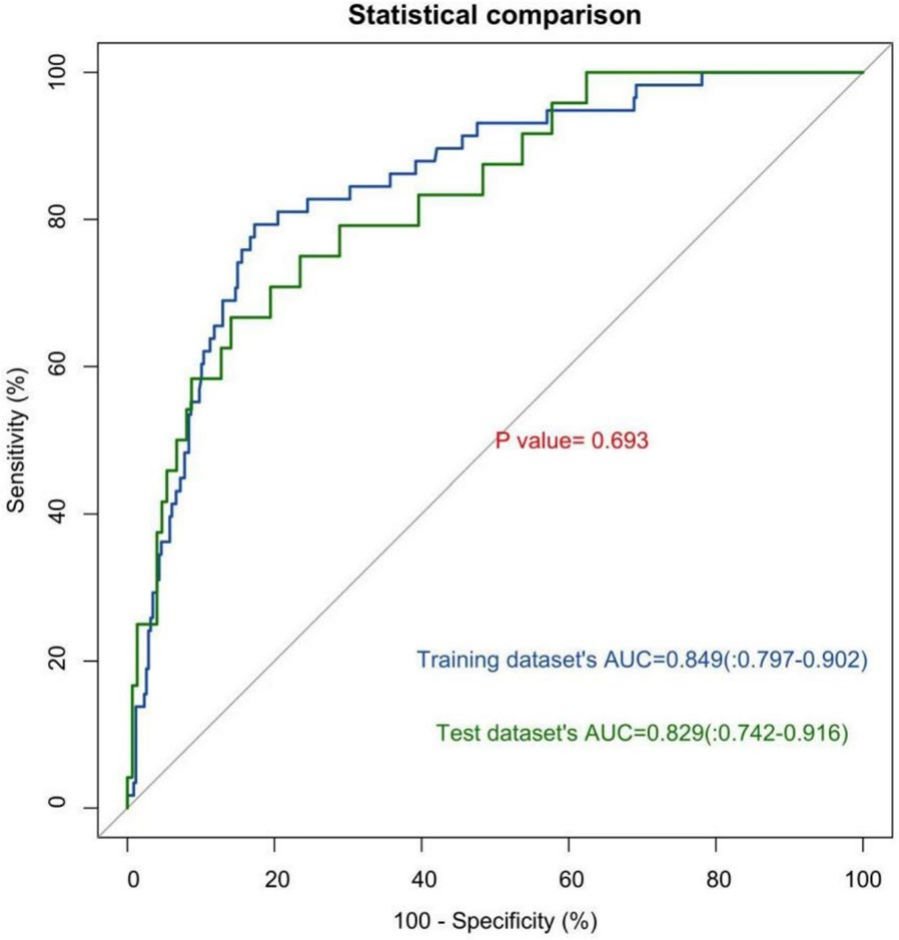
ROC curve graph for model with training data set and test data set

Leveraging multivariate logistic regression analysis, a nomogram model was constructed using R software to quantitatively evaluate the risk of stroke progression in patients (Fig. 2). Each index corresponds to a score in the top point line, and then the total point score is the sum of the four index scores. The total point score is projected on the bottom scales to judge the probability of PIS after AIS patients. The diagnostic performance of the nomogram was evaluated using receiver operating characteristic (ROC) curves for both the training and test data sets (Table 3). As shown in Fig. 3, the area under the curve (AUC) values were 0.849 (95% CI 0.797–0.902) for the training data set and 0.829 (95% CI 0.742–0.916) for the test data set. Although the AUC for the training data set was slightly higher than that of the test data set, the *DeLong* test revealed no statistically significant difference between the two (χ^2^ = 0.395, *P* = 0.693 > 0.05), indicating consistent performance across both data sets.

**Table 3:** Comparison of the effect indicators for the different models

Groups	AUC(95%CI)	Sensitivity	Specificity	Precision (95% CI)
Training data set	0.849(0.797,0.902)	0.724	0.850	0.832(0.792,0.867)
Test data set	0.829(0.742,0.916)	0.667	0.859	0.832(0.768,0.885)

**Fig. 4:**
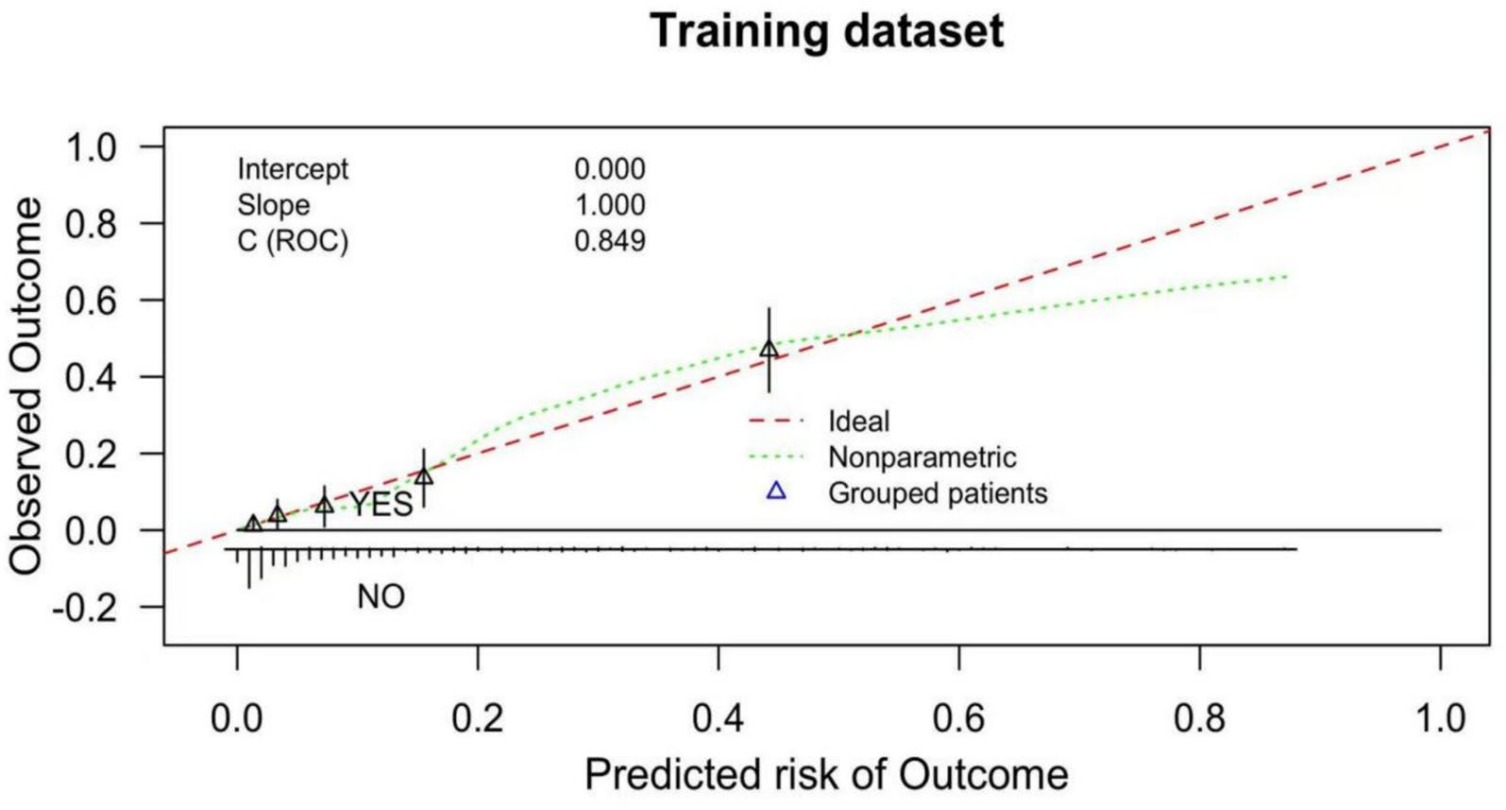
Calibration curve of the prediction model in the training data set

**Fig. 5:**
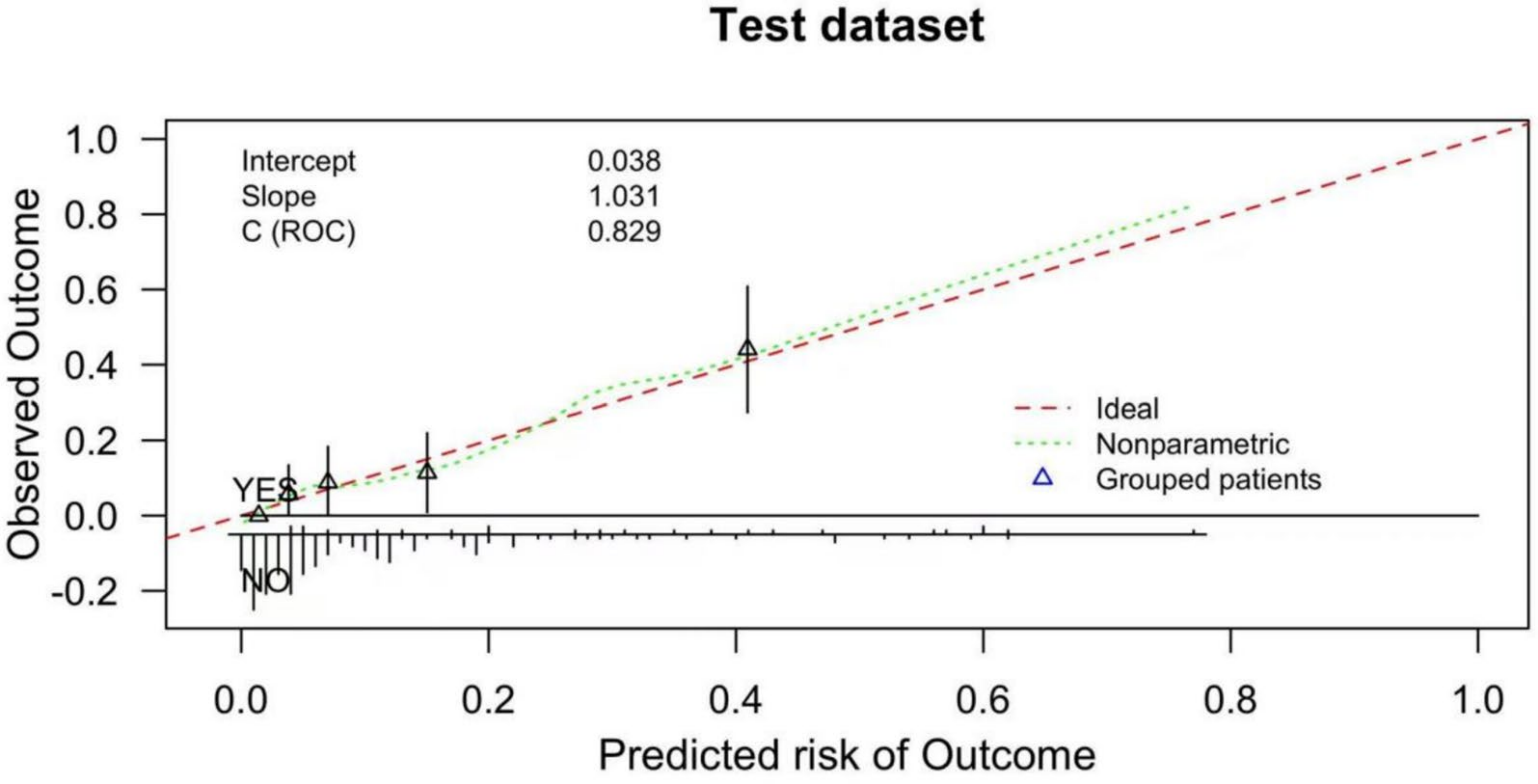
Calibration curve of the prediction model in the test data set

The calibration of the nomogram model was further assessed to evaluate the agreement between predicted risks and actual outcomes. In the training data set, the calibration curve demonstrated strong concordance, as confirmed by the Hosmer–Lemeshow test (*χ*^*2*^ = 6.831, *P* = 0.555; Fig. 4). Similarly, in the test data set, the model exhibited comparable agreement between predicted and observed outcomes, supported by the Hosmer–Lemeshow test (*χ*^*2*^ = 4.805, *P* = 0.778; Fig. 5). These findings underscore the robustness and reliability of the nomogram model in predicting stroke progression.

### The nomogram model demonstrated superior clinical applicability

To comprehensively evaluate the clinical utility of the nomogram model in both the training and test data sets, DCA was employed to assess the net benefit across varying Probability threshold (Pt). DCA quantifies the clinical benefit by comparing the trade-off between true-positive identifications and false-positive results for each model or indicator at different Pt values. This approach determines the optimal proportion of PIS cases that can be identified among cerebral infarction patients, relative to scenarios, where no neurological examination is performed while minimizing unnecessary additional testing.

**Fig. 6:**
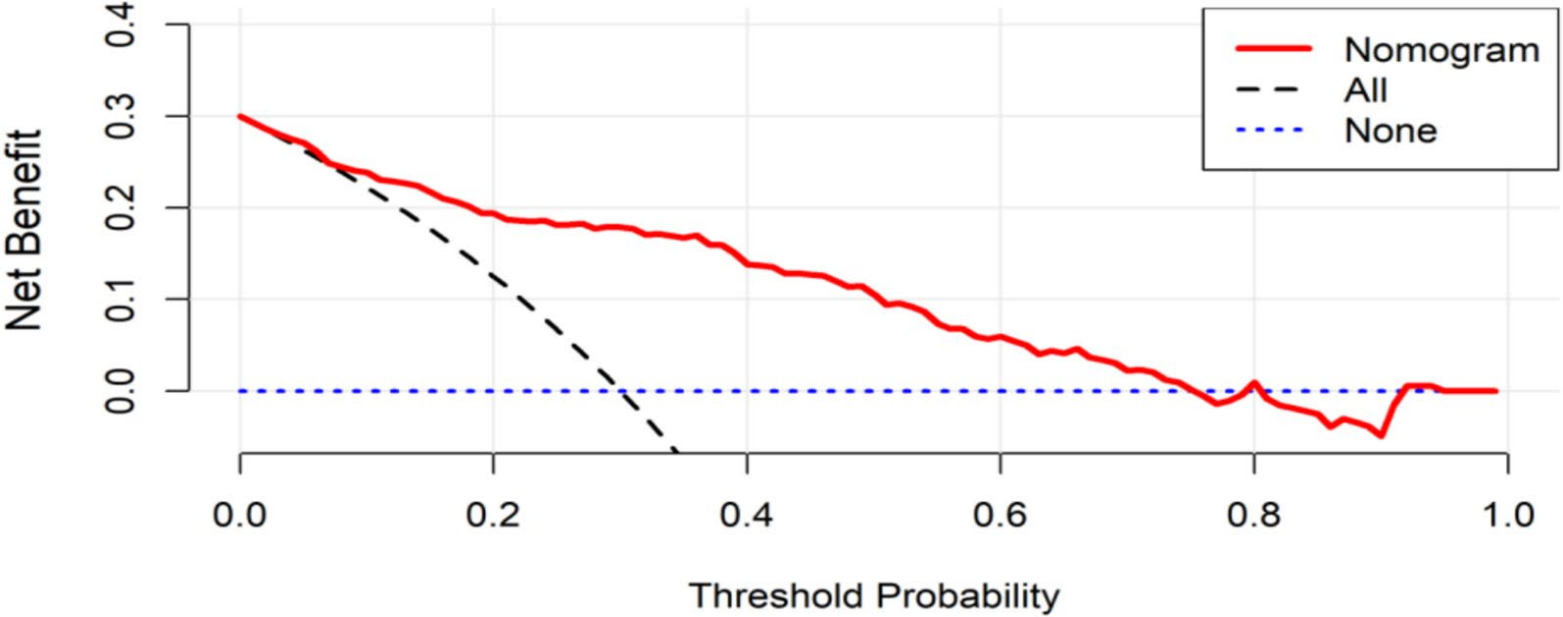
Results of the DCA analysis of the prediction model in the training data set

**Fig. 7:**
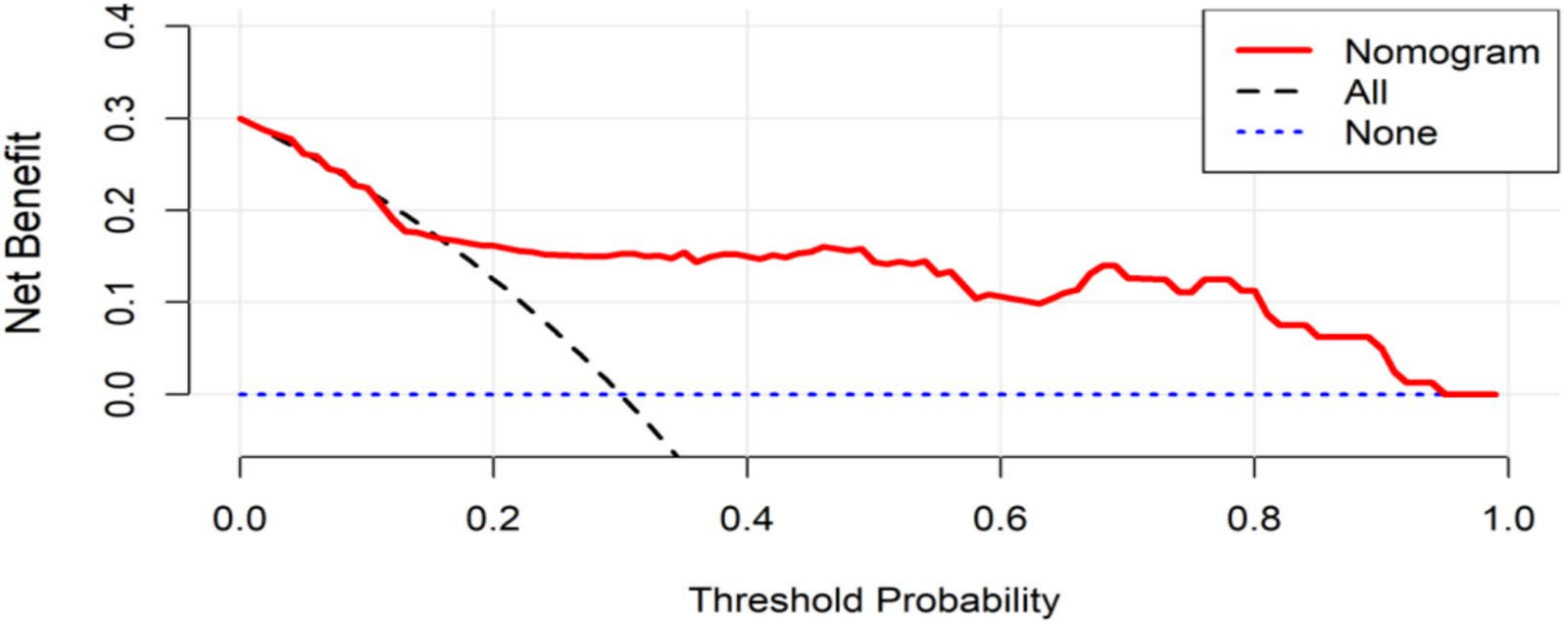
Results of the DCA analysis of the prediction model in the test data set

As illustrated in Figs. 6 and 7, the constructed nomogram prediction Model demonstrated a progressive increase in net benefit when the Pt was set at or above 10%. In practical clinical settings, adopting a Pt value of 10% enables the detection of an additional 10 PIS cases per 100 screened individuals without significantly increasing the burden of unnecessary examinations or the rate of false positives. This underscores the model's ability to enhance diagnostic efficiency while maintaining clinical relevance.

## Discussion

This study was designed to identify predictors of PIS and to develop a practical nomogram that incorporates these predictors. Our analysis identified diabetes mellitus, LAA, Lp-PLA2, and age as independent risk factors for PIS. Importantly, the nomogram, which integrates LAA and Lp-PLA2 along with other critical predictors, exhibited robust predictive accuracy and substantial clinical utility for assessing the risk of PIS. This tool offers the potential to enhance the early identification of patients at increased risk for PIS, thereby facilitating more informed clinical decision-making and the formulation of tailored preventive strategies.

The pathogenesis of PIS remains incompletely understood, encompassing a variety of pathophysiological mechanisms. The primary mechanisms implicated include hemodynamic changes, systemic factors, and inflammatory processes. Risk factors for PIS are generally classified into broad and specific categories. Broad risk factors include age, lifestyle habits, metabolic syndrome, obesity, hypersensitivity C-reactive protein, high fibrinogen levels, and alterations in these markers, which can influence clinical outcomes in PIS patients, though they lack specificity [14, 15]. Specific risk factors include blood pressure, blood glucose levels, homocysteine levels, site of infarction, NIHSS score, atherosclerosis, presence of microemboli, aspirin resistance, among others [16]. Research within the TOAST classification indicates that PIS associated with large artery atherosclerotic cerebral infarction presents an elevated risk [17, 18], necessitating vigilant clinical monitoring. Furthermore, an international study has demonstrated that a CHA2DS2–VASc score of 5 at admission correlates significantly with the risk of PIS in patients experiencing cardiogenic stroke due to atrial fibrillation, particularly in those with intracranial atherosclerosis [19]. Notably, the atherosclerotic cerebral infarction type and diabetes mellitus align with the findings of this study. It is plausible to suggest that the incidence of progressive stroke is higher in cases of arteriosclerotic cerebral infarction, potentially related to hemodynamic disturbances and the inflammatory responses triggered by plaque rupture [20]

Lp-PLA2 functions as a serum biomarker that reflects the stability of atherosclerotic plaques and has been associated with adverse stroke outcomes and an increased risk of subsequent vascular events in previous studies [21–23]. The exact mechanisms underlying this association have not yet been fully elucidated. It is hypothesized that Lp-PLA2, secreted by inflammatory cells within atherosclerotic plaques, exacerbates inflammation by generating proinflammatory substances, thereby contributing to endothelial dysfunction, plaque inflammation, and the formation of necrotic cores [24]. Furthermore, elevated levels of Lp-PLA2 have been identified as indicative of an atherogenic profile and an independent predictor of cerebral infarction progression [25], corroborating our findings.

Studies have shown that chronic inflammation has been associated with an increased burden of atherosclerotic disease and an elevated risk of ischemic stroke [26, 27]. Lp-PLA2 are related to the inflammatory response after ischemic stroke [28]. Lp-PLA2, an enzyme derived from leukocytes, is released in response to inflammation and is believed to directly promote atherogenesis. It does so by hydrolyzing low-density lipoprotein (LDL) to produce oxidized phospholipids, which possess inherent inflammatory properties and trigger proinflammatory downstream signaling [29]. The prevailing hypothesis suggests that Lp-PLA2 hydrolyzes oxidized phospholipids, producing proinflammatory byproducts implicated in endothelial dysfunction, plaque inflammation, and the formation of necrotic cores within plaques. This enzyme is postulated to connect the oxidative modification of LDL with the development of inflammatory responses in the arterial intima [30]. Inflammatory markers have been consistently reported to correlate with the recurrence of vascular events [31]. Elevated levels of Lp-PLA2 may enhance platelet counts, potentially intensifying platelet–endothelial interactions, inflammation, and plaque rupture. This sequence of events can promote platelet adhesion, aggregation, and activation, thereby increasing the likelihood of thrombosis as well as the risk of recurrent and progressive stroke [32–34].

Moreover, thrombosis and inflammation are intricately connected in most conditions associated with ischemia-induced organ damage [35, 36].Lp-PLA2 has been implicated in vascular inflammation-related diseases by facilitating macrophage migration and activation [37, 38], which may influence inflammatory pathways, accelerating thrombus formation and ultimately resulting in stroke recurrence and progression. It is evident from the preceding discussion that LpPLA2 may engage with additional risk factors, particularly in individuals experiencing hyperglycemia and hyperlipemia, which together may contribute to the advancement of vascular diseases.

In summary, a synthesis of prior research confirms that variables such as atherosclerotic stroke subtype, diabetes, age, and systemic inflammatory status independently correlate with the incidence of PIS in patients with AIS. This study corroborates these findings, establishing LAA, T2DM, and age as robust independent predictors of PIS. Furthermore, our research identifies Lp-PLA2 as an independent factor linked to PIS and explores its potential molecular biological mechanisms involving inflammation and lipid metabolism. In this study, we introduce and evaluate a new nomogram risk prediction model for PIS in AIS patients, which incorporates factors, such as LAA, T2DM, age, and Lp-PLA2. Preliminary findings suggest that this nomogram may offer substantial predictive accuracy and clinical net benefit in assessing the risk of PIS.

Existing predictive models for PIS in patients with AIS are limited. Recent investigations, including those conducted by Yingying Wang and colleagues, have identified the leukocyte-to-monocyte ratio (LMR) as an independent predictor of PIS in this cohort. These studies have also demonstrated that integrating LMR with the NIHSS significantly enhances the prediction of adverse long-term outcomes in AIS patients [39]. Contrasting with the study by Yingying Wang, which exclusively examined the predictive value of systemic inflammatory status for PIS, our research adopts a more holistic approach. We have developed a risk prediction model for PIS by integrating multiple independent factors, including age, stroke subtype, medical history, and hematological markers. This model seems to offer enhanced predictive power and stability compared to single indicators, potentially providing greater clinical benefit. It may assist physicians in identifying high-risk patients who could benefit from closer monitoring, more stringent control measures, and frequent reassessment. Furthermore, this tool might encourage patients to engage in proactive lifestyle modifications and adhere to preventive pharmacotherapy.

This study has several notable limitations that warrant discussion. First, its design as a single-center retrospective study inherently limits the breadth of data and may not accurately reflect a broader patient population. The relatively small sample size further constrains the statistical power and the robustness of our findings. Consequently, to enhance our understanding of the independent factors associated with the occurrence of PIS following AIS, as well as to assess the stability and generalizability of the developed nomogram risk prediction model, extensive validation is essential. Future studies should aim to replicate our findings in multicenter trials that include larger and more diverse patient cohorts. Second, our results suggest that the nomogram risk prediction model, which incorporates large artery atherosclerosis LAA, T2DM, age, and Lp-PLA2, demonstrates high predictive performance for PIS in AIS patients. However, the potential therapeutic impact of anti-inflammatory and lipid-lowering treatments on the modifiable risk factor Lp-PLA2 remains uncertain. It is unclear whether such interventions can effectively delay the occurrence of PIS in this patient group. Therefore, there is a pressing need for prospective, large-scale studies that explore the effectiveness of anti-inflammatory interventions and lipid correction in AIS patients to determine their role in preventing PIS. Finally, while this study focused on the predictive performance of the nomogram risk prediction model for the occurrence of PIS in AIS patients, the long-term predictive value of this model for adverse outcomes in PIS patients has not been investigated. Future research should extend beyond the immediate risk prediction to explore the long-term outcomes associated with PIS, thereby providing a more comprehensive tool for clinical decision-making in the management of AIS patients.

## Conclusions

Lp-PLA2 has been identified as an independent predictor of PIS. Notably, patients with elevated Lp-PLA2 levels at emergency admission, especially older adults with T2DM and a predisposition to arteriosclerotic cerebral infarction, exhibited a significantly increased risk of PIS. While Lp-PLA2 alone showed modest sensitivity and specificity in predicting PIS among patients with AIS, the inclusion of Lp-PLA2 in a nomogram model alongside LAA enhanced predictive accuracy substantially. This model demonstrates considerable clinical utility, offering potential for the identification of high-risk individuals and informing more personalized preventive strategies for PIS.

## Data Availability

Data from this study are available from the corresponding author upon request.

## Declarations

**Ethical approval and informed consent**

This study was conducted in accordance with the Declaration of Helsinki and was approved by the Local Ethics Committee of the Affiliated Hospital of Xuzhou Medical University (ethics approval number XYFY2019-KL042-01). Considering the retrospective nature of the study, the requirement for patient consent was waived by the ethics committee. All patient data were anonymized or maintained confidentially.
